# Differential Pathogenesis of SARS-CoV-2 Variants of Concern in Human ACE2-Expressing Mice

**DOI:** 10.3390/v14061139

**Published:** 2022-05-25

**Authors:** Janhavi Prasad Natekar, Heather Pathak, Shannon Stone, Pratima Kumari, Shaligram Sharma, Tabassum Tasnim Auroni, Komal Arora, Hussin Alwan Rothan, Mukesh Kumar

**Affiliations:** Department of Biology, College of Arts and Sciences, Georgia State University, Atlanta, GA 30303, USA; jnatekar1@gsu.edu (J.P.N.); hpathak1@gsu.edu (H.P.); sstone12@student.gsu.edu (S.S.); pkumari1@gsu.edu (P.K.); ssharma17@student.gsu.edu (S.S.); tauroni1@student.gsu.edu (T.T.A.); karora@gsu.edu (K.A.); hrothan@gsu.edu (H.A.R.)

**Keywords:** COVID-19, SARS-CoV-2 variants, omicron, ACE2-expressing mice, inflammation

## Abstract

Severe acute respiratory syndrome coronavirus 2 (SARS-CoV-2) has caused the current pandemic, resulting in millions of deaths worldwide. Increasingly contagious variants of concern (VoC) have fueled recurring global infection waves. A major question is the relative severity of the disease caused by previous and currently circulating variants of SARS-CoV-2. In this study, we evaluated the pathogenesis of SARS-CoV-2 variants in human ACE-2-expressing (K18-hACE2) mice. Eight-week-old K18-hACE2 mice were inoculated intranasally with a representative virus from the original B.1 lineage or from the emerging B.1.1.7 (alpha), B.1.351 (beta), B.1.617.2 (delta), or B.1.1.529 (omicron) lineages. We also infected a group of mice with the mouse-adapted SARS-CoV-2 (MA10). Our results demonstrate that B.1.1.7, B.1.351 and B.1.617.2 viruses are significantly more lethal than the B.1 strain in K18-hACE2 mice. Infection with the B.1.1.7, B.1.351, and B.1.617.2 variants resulted in significantly higher virus titers in the lungs and brain of mice compared with the B.1 virus. Interestingly, mice infected with the B.1.1.529 variant exhibited less severe clinical signs and a high survival rate. We found that B.1.1.529 replication was significantly lower in the lungs and brain of infected mice in comparison with other VoC. The transcription levels of cytokines and chemokines in the lungs of B.1- and B.1.1.529-infected mice were significantly less when compared with those challenged with other VoC. Together, our data provide insights into the pathogenesis of previous and circulating SARS-CoV-2 VoC in mice.

## 1. Introduction

SARS-CoV-2 is a positive-sense, single-stranded RNA virus belonging to the Betacoronavirus family [1,2]. Since the emergence of SARS-CoV-2 in late 2019, several new variants of concern (VoC), alpha (B.1.1.7 lineage), beta (B.1.351 lineage), gamma (P.1 lineage), delta (B.1.617.2 lineage), and omicron (B.1.1.529 lineage), have fueled recurring global infection waves. These variants have been termed VoC because of the higher risk due to their possible enhanced transmissibility, disease severity, immune escape, and increased adaptation to new hosts [3,4,5,6,7,8]. Mutations occurring in the spike protein are of major concern due to the role of this glycoprotein in mediating virus entry and as the major target of neutralizing antibodies [3,9,10,11]. The lineage B.1.1.7 was first identified in the United Kingdom, lineage B.1.351 was discovered in South Africa, and lineage B.1.617.2 was first described in India. Most recently, the omicron (B.1.1.529) VoC that emerged in South Africa was estimated to have been responsible for the majority of infections worldwide. The B.1.1.7 variant has mutations in the receptor binding domain (RBD) region, including N501Y, 69/70 deletion, and P681H near the S1/S2 furin cleavage site [7,12,13,14]. The B.1.351 variant has eight mutations, of which the three most notable mutations are K417N, E484K, and N501Y in the spike protein [3,7,9,15]. The B.1.617.2 variant has three unique mutations: E156del/R158G in the N-terminal domain and T478K in RBD of the spike protein. The B.1.1.529 variant has an unusually large number of mutations in the spike protein, including 30 amino acid substitutions, three short deletions, and one insertion [16,17,18].

The main goal of this study was to compare the replication and pathogenesis of SARS-CoV-2 variants in K18-hACE2 mice. K18-hACE2 is a transgenic mouse model that expresses human ACE2 driven by the human cytokeratin 18 promoter. K18-hACE2 mice is a well-established model for SARS-CoV-2 studies that supports virus replication in the respiratory and central nervous systems, resulting in elevated chemokine and cytokine levels, and significant tissue pathologies [19,20,21]. Our results demonstrate that the B.1.1.7 (alpha), B.1.351 (beta), and B.1.617.2 (delta) variants are more virulent than the original SARS-CoV-2 B.1 Wuhan strain in K18-hACE2 mice. Infection with the B.1.1.7, B.1.351, and B.1.617.2 variants resulted in significantly high virus titers in the lungs and brain of mice compared with the B.1 virus. Interestingly, the replication capacity of the omicron variant was significantly lower than other VoC. Mice infected with the B.1.1.529 virus exhibited high survival rate and had a lower virus load in the lungs and brain compared with mice infected with the B.1.1.7, B.1.351, and B.1.617.2 viruses. In addition, B.1- and B.1.1.529-infected mice had significantly attenuated inflammation in the lungs compared with those inoculated with other VoC.

## 2. Materials and Methods

### 2.1. In Vivo Mouse Challenge Experiments

In vivo mouse experiments involving infectious SARS-CoV-2 were performed in Animal Biosafety Level 3 laboratory and strictly followed the approved standard operation procedures. The protocol was approved by the Georgia State University Institutional Animal Care and Use Committee (Protocol number A20044). Hemizygous K18-hACE2 mice (2B6.Cg-Tg (K18-ACE2)2Prlmn/J) were obtained from The Jackson Laboratory. Eight-week-old hemizygous K18-ACE2 mice were inoculated intranasally with PBS (mock) or 10^4^ plaque-forming units (PFU) of SARS-CoV-2, as described previously [8,21,22]. We used the B.1 Wuhan virus (BEI# NR-52281), B.1.1.7 virus (BEI# NR-54000), B.1.351 virus (BEI# NR-54008), B.1.617.2 virus (Northwestern Reference laboratory, Clinical isolate #2333067), B.1.1.529 virus (BEI# NR-56461), and MA10 virus (BEI# NR-55329). Roughly equal numbers of male and female mice were used. The animals were weighed, and their appetite, activity, breathing, and neurological signs were assessed twice daily. In independent experiments, mice were inoculated with PBS (Mock) or SARS-CoV-2 intranasally, and on days 3 and 5–7 after infection, the animals were anesthetized using isoflurane and perfused with cold PBS. The lungs and brain were collected and flash-frozen in 2-methylbutane (Sigma, St. Louis, MO, USA) for further analysis, as described below [8,23]. 

### 2.2. Infectious Virus Titration by Plaque Assay

Tissues harvested from virus-inoculated animals were weighed and homogenized in a bullet blender (Next Advance, Averill Park, NY, USA) using stainless steel or zirconium beads, followed by centrifugation and titration. Virus titers in tissue homogenates were measured by plaque assay using Vero E6 cells [8]. To titrate the infectious virus, tissue homogenates were 10-fold serially diluted with DMEM and applied to monolayered Vero E6 cells for 1 h. After inoculation, the cells were washed once before overlaid with 1% low-melting agarose. The cells were further incubated for 48 h and stained with neutral red for visualizing plaque formation [8,24].

### 2.3. RNA Extraction and Quantitative RT-PCR

Total RNA was extracted from the lungs using a Qiagen RNeasy Mini kit (Qiagen, Germantown, MD, USA). The cDNA was synthesized from RNA using an iScript™ cDNA Synthesis Kit (Bio-Rad). The qRT-PCR was used to determine the expression levels of IL-6 and CCL-2, as described previously [22,24]. The fold-change in infected samples compared with control samples was calculated after normalizing to the housekeeping GAPDH gene [22,23]. The primer sequences used for qRT-PCR are listed in Table 1.

### 2.4. Statistical Analysis

GraphPad Prism 8.0 was used to perform a Kaplan–Meier log-rank test to compare survival curves. For body weight changes, two-way analysis of variance (ANOVA) with the post hoc Bonferroni test was used to calculate the *p* values. The Mann–Whitney test and unpaired student *t*-test were used to calculate the *p* values of the difference between viral titer and immune responses, respectively. Differences of *p* < 0.05 were considered significant.

## 3. Results

### 3.1. Clinical Disease Progression of K18-hACE2 Mice Infected with SARS-CoV-2 VoC

**Figure 1:**
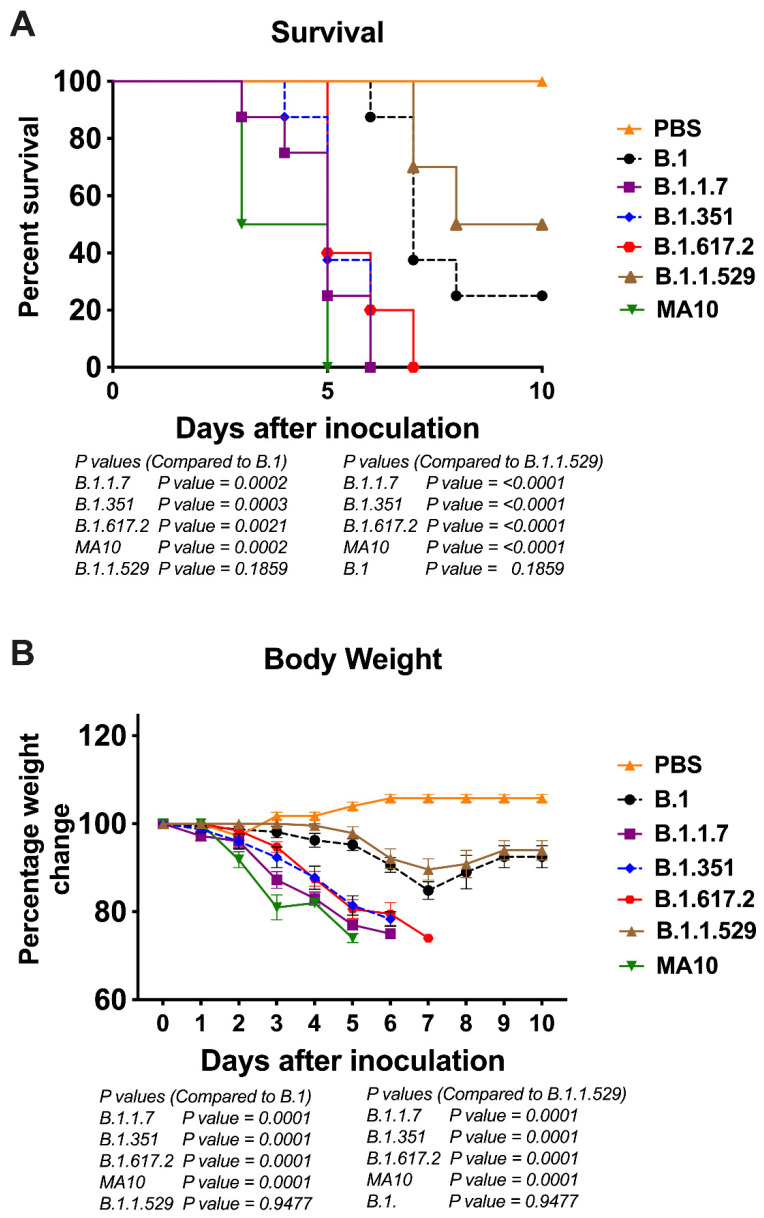
Analysis of survival and body weight in K18-hACE2 mice following infection with SARS-CoV-2 VoC. Eight-week-old K18-hACE2 mice were inoculated intranasally with PBS (mock) or 10^4^ PFU of B.1 and individual variants (*n* = 10–15 mice per group). (**A**) Survival curve. (**B**) Body weight change in percentage. Values are the mean ± SEM.

To evaluate the pathogenicity of the original B.1 lineage and emerging SARS-CoV-2 VoC derived from the B.1 lineage, K18-hACE2 mice were infected intranasally with a representative virus from the original B.1 lineage or the emerging B.1.1.7 (alpha), B.1.351 (beta), B.1.617.2 (delta), and B.1.1.529 (omicron) lineages. We also infected a group of mice with the mouse-adapted SARS-CoV-2 (MA10) [25]. The animals were monitored for clinical signs and survival. The mock-infected mice remained healthy throughout the study period. While infectious doses of 10^4^ plaque-forming units (PFU) of B.1 virus resulted in 75% mortality, mortality in the B.1.1.7-, B.1.351-, and B.1.617.2-infected mice was 100% (Figure 1A). The median survival times of mice infected with the alpha, beta, and delta variants were also shorter than those in the B.1-infected mice. Statistically, mouse survival for the B.1 virus was significantly higher than the alpha, beta, and delta variants. K18-hACE2 mice infected with the MA10 virus showed a faster disease progression and severity after infection compared with all SARS-CoV-2 clinical isolates. Interestingly, infection with the B.1.1.529 (omicron) virus resulted in only 50% mortality with extended survival time in mice (Figure 1A). There was a significant difference between the survival of the B.1.1.529 challenged mice compared with the other VoC at the same dose. 

As early as 3 days after infection, mice inoculated with the B.1.1.7 or B.1.351 virus began to lose body weight and showed signs of infection. By 6 days, all mice infected with the B.1.1.7 or B.1.351 virus died after losing 20% body weight and experiencing severe symptoms (Figure 1B). Mice infected with the B.1.617.2 also lost significant body weight, and all succumbed to death by day 7 after infection. Statistically, body weight loss for the B.1.1.7, B.1.351, and B.1.617.2 viruses was significantly higher than that for the B.1 virus infections. Compared with the other VoC, a body weight loss of mice infected by the B.1.1.529 virus was significantly milder with onset time at a later stage during the infection (Figure 1B). 

### 3.2. Viral Load in K18-hACE2 Mice Infected with SARS-CoV-2 VoC

**Figure 2:**
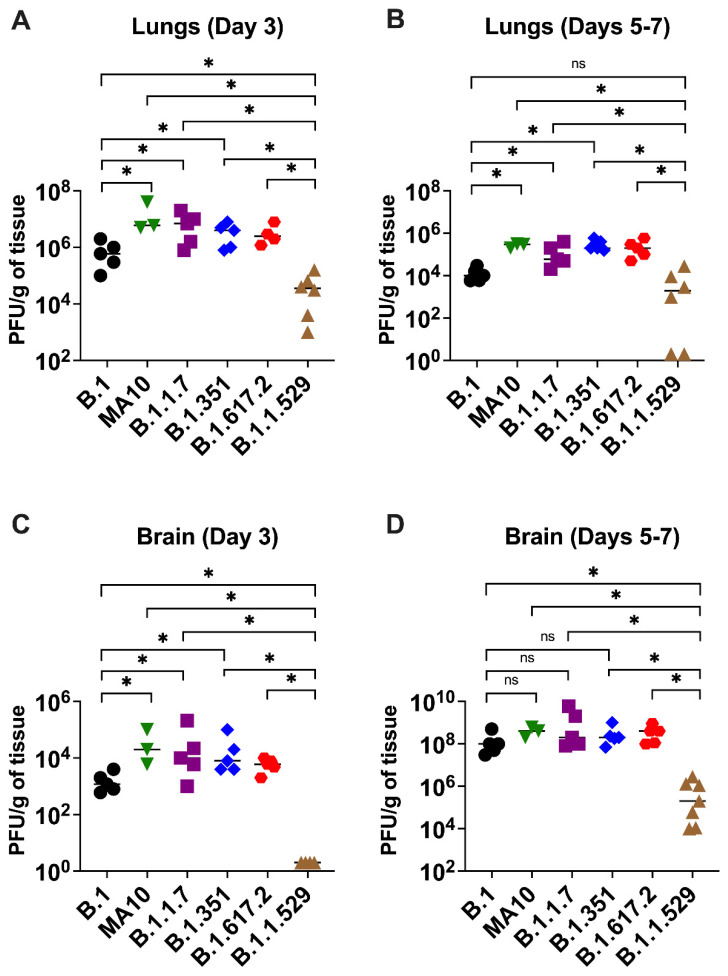
Replication of SARS-CoV-2 VoC in the lungs and brain. Eight-week-old K18-hACE2 mice were inoculated intranasally with 10^4^ PFU of SARS-CoV-2 or variants. Groups of 3–7 mice were euthanized on day 3 and days 5–7 after infection, and tissues were collected. Virus titers were analyzed in the lungs and brain by plaque assay. The data are expressed as PFU/g of tissue. (**A**) Virus titer in day 3 lungs, (**B**) virus titer in day 5–7 lungs, (**C**) virus titer in day 3 brain, and (**D**) virus titer in day 5–7 brain. Each data point represents an individual mouse. *, *p* < 0.05.

To evaluate virus replication in the tissues, groups of 3–7 mice were euthanized at 3- and 5–7 days after infection, and the lungs and brain were collected. Viral infectivity titer in the tissues were measured by a plaque assay. A median infectious virus titer of 5 × 10^5^ PFU/g was detected at day 3 after infection in the lungs from the animals infected with the B.1 virus. Compared with the B.1 virus, infections with B.1.1.7, B.1.351, and B.1.617.2 viruses resulted in significantly higher levels of infectious virus in the lungs at day 3 after infection (Figure 2A). On days 5–7 after infection, mice infected with the B.1.1.7, B.1.351, and B.1.617.2 viruses sustained significantly high levels of viral load in the lungs compared with the B.1 virus (Figure 2B). In contrast, the replication of the B.1.1.529 virus was dramatically reduced in comparison with those of B.1.1.7, B.1.351, and B.1.617.2 viruses, despite using the same inoculation titer for virus challenge. The level of infectious virus in the lungs of B.1.1.529-infected mice was approximately 100-fold lower than in the animals infected with other VoC at both 3 and 5–7 days after infection (Figure 2A,B). 

In the brain, mice infected with the B.1 virus exhibited significantly lower levels of infectious virus than B.1.1.7-, B.1.351-, and B.1.617.2-infected animals at day 3 after infection (Figure 2C). At 5–7 days after infection, the viral loads were similar in animals infected with the B.1, B.1.1.7, B.1.351, and B.1.617.2 viruses (Figure 2D). Consistent with the lung data, the infectious virus in the brain harvested from the B.1.1.529-infected mice was also significantly reduced compared with that of the other groups. We did not detect any infectious virus in the B.1.1.529-infected mice at day 3 after infection. On 5–7 days after infection, the viral load was approximately 1000-fold higher in the B.1.1.7-, B.1.351-, and B.1.617.2-infected mice than the B.1.1.529-infected mice (Figure 2C,D). We did not detect any infectious virus in the lungs and brain of B.1 and B.1.1.529-infected mice that survived the infection. A relative decrease in viral replication of the B.1 and B1.1.529 lineages when comparing the data from lungs and brain after 3 days and 5–7 days of infection (Figure 2) was correlated with the observed decline in clinical and pathological severity and recovery seen in infected mice in these groups (Figure 1).

### 3.3. Inflammation in the Lungs following Infection with SARS-CoV-2 VoC

**Figure 3:**
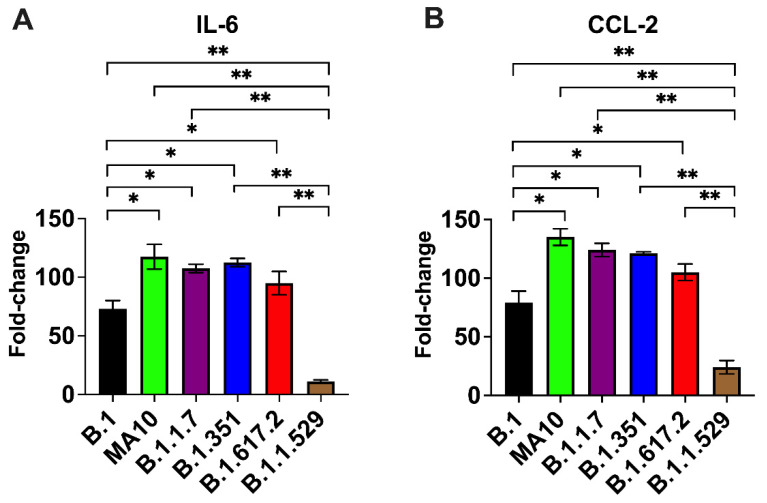
Analysis of inflammatory response in the lungs. Eight-week-old K18-hACE2 mice were inoculated intranasally with PBS (mock) or 10^4^ PFU of SARS-CoV-2 or variants (*n* = 5–7 mice per group). Lungs and brain were harvested after extensive perfusion with PBS and RNA was extracted. The mRNA levels of (**A**) IL-6 and (**B**) CCL-2 were determined by qRT-PCR. Values are the mean ± SD. *, *p* < 0.05, **, *p* < 0.001.

The excessive inflammatory host response to SARS-CoV-2 infection contributes to pulmonary pathology and the development of respiratory distress in patients infected with COVID-19 [26,27]. We next quantified the gene expression of IL-6 and CCL-2 in the lungs of K18-hACE2 mice at day 3 after infection. Gene expression changes in the lungs of infected mice, compared with the mock-infected controls, were analyzed after normalizing each sample to the level of the endogenous GAPDH gene. We did not find any significant change in the expression level of the GAPDH gene as a consequence of viral inflammation. As shown in Figure 3, infections with the B.1.1.7, B.1.351, B.1.617.2, and MA10 viruses resulted in >100-fold increases in IL-6 and CCL-2 mRNA expression. In contrast, the IL-6 and CCL-2 mRNA levels increased by 60–75-fold in the lungs of the B.1-infected mice. Mice inoculated with the omicron variant had the lowest mRNA levels of IL-6 and CCL-2 (10–20-fold) compared with those inoculated with other VoC and the B.1 virus, suggesting attenuated inflammation (Figure 3).

## 4. Discussion

To date, most K18-hACE2 mouse studies have utilized the original viral strains of SARS-CoV-2 and few studies have been performed with emergent VoC. In this study, we investigated the pathogenicity of emerging SARS-CoV-2 VoC derived from the B.1 lineage in K18-hACE2 mice. Our results demonstrate that the pathogenicity of SARS-CoV-2 in K18-hACE2 mice is VoC-dependent and the highest for the alpha, beta, and delta variants. We found significantly higher virus titers in the lungs and brain of mice infected with the B.1.1.7, B.1.351, and B.1.617.2 variants compared with the B.1 lineage. In contrast, the omicron variant replicated significantly less efficiently than other SARS-CoV-2 variants in mice. In comparison with the alpha, beta, and delta variants, the omicron variant results in less body weight loss and a lower mortality rate. This is also reflected by less extensive inflammatory processes in the lungs of the B.1.1.529-infected mice. 

SARS-CoV-2 evolves rapidly with the accumulation of mutations in the viral genome, giving rise to multiple variants of concern (VoC) [4,5,10]. Among all mutations, N501Y is the most critical because it involves amino acid residues that account for the tight binding of RBD of the SARS-CoV-2 and ACE2 receptors on the host cell surface [7,10,11]. The initial strains of SARS-CoV-2 had spike proteins unable to utilize mouse ACE2 and infect standard laboratory mice. To overcome this barrier, a mouse-adapted SARS-CoV-2 variant (MA10) with binding affinity to mouse ACE-2 was obtained after sequential passaging of virus in mouse lung tissues [25,28]. The MA10 virus causes infection, inflammation, and pneumonia in BALB/c mice after intranasal inoculation. MA10 has several mutations, including the N501Y mutation in the RBD of the spike protein compared with the Wuhan reference sequence [25,28]. B.1.1.7 and B.1.351 lineages contain N501Y in RBD in addition to widespread D614G in a spike protein, while the B.1.351 variant also has two additional mutations: K417N and E484K [10,12,13,15]. Both N501Y and D614G have been shown to increase RBD binding to ACE2 and to promote virus entry and replication in humans and animal models [3,7,8,9,10,11]. These mutations in the RBD of the spike protein may have enhanced the binding affinity for the ACE2 receptor, thereby allowing the variants to replicate more efficiently in mice. Indeed, our results demonstrate that SARS-CoV-2 variants B.1.1.7 and B.1.351 containing N501Y and E484K mutations display a substantially severe pathogenicity in K18-hACE2 mice. B.1.1.7 and B.1.351 caused lethal disease in K18-hACE2 mice accompanied with higher-titer viral burden in the lungs and brain. The B.1.617.2 (delta) lineage does not harbor the N501Y substitution in the spike protein but has additional mutations within the spike protein that diverge from other VoC. In the B.1.617.2 lineage, two modifications, namely L452R and T478K in the RBD, increase the interaction with ACE2 with the highest binding affinity [29,30], which may contribute to an increase in pathogenicity observed in mice. 

Our results corroborated the recent findings that the alpha, beta, and delta variants were able to cause enhanced disease in K18-hACE2 mice [6,14,31,32]. In the present study, we did not conduct a comprehensive transcriptome analysis of mice lungs and brain that were infected with SARS-CoV-2 variants. However, other recent studies have shown that intranasal infection of K18-hACE2 mice with B.1.1.7 and B.1.351 variants result in distinct tissue-specific proinflammatory cytokine signatures and a lack of extensive pulmonary hypoxia signaling before death. Several myeloid cell chemo-attractants showed enhanced pulmonary secretions in K18-hACE2 mice following exposure to the B.1.1.7 or B.1.351 variants [6,31]. It remains unclear whether these distinct cytokine profiles are due to enhanced replication of the B.1.1.7 and B.1.351 viruses in mice or are caused by specific mutations in these variants. Recent studies have also shown reduced leukocyte infiltration to the lungs during B.1.617.2 infection, suggesting that this variant may have a novel mechanism to evade immune response [32]. 

Despite carrying the highest number of mutations that might allow for more efficient binding to ACE2, we observed mild disease in mice infected with the omicron variant compared with other SARS-CoV-2 variants. Epidemiological data also suggest that the omicron virus causes a less severe pathology in humans than the ancestral strains and the other VoC [29]. Our results agree with recent studies that demonstrated attenuated replication of the omicron variant in mice and hamsters [16,18]. Recently, in vitro studies have shown that the omicron variant is inefficient in transmembrane serine protease 2 (TMPRSS2) usage in comparison with that of previous variants [33,34]. It is possible that the attenuated replication of the omicron variant in human cells and mice is due to a reduced efficiency in host protease cleavage by changes in the spike protein. However, other species-specific factors may play a role in attenuated pathogenicity of the omicron variant observed in hACE2 mice. One possibility is that the omicron variant uses alternative (ACE2-independent) infection routes and that hACE2-expressing mice are a less effective model for this variant. Overall, our study demonstrates that SARS-CoV-2 pathogenicity in K18-hACE2 mice is VoC-dependent; is the highest for the alpha, beta, and delta variants; and is the lowest for the omicron variant. These results will be valuable for understanding the pathogenesis of emerging SARS-CoV-2 variants.

## Figures and Tables

**Table 1:** Primer sequences used for qRT-PCR.

Gene (Accession No.)	Primer Sequence (5′–3′)
GAPDH (NM_008084) Forward Reverse IL-6 (NM_000600)	CAGTATGACTCCACTCAC GTAGACTCCACGACATAC
Forward	CCAGGAGCCCAGCTATGAAC
Reverse	CCCAGGGAGAAGGCAACTG
CCL2 (NM_011333)	
Forward	TCACCTGCTGCTACTCATTCACCA
Reverse	TACAGCTTCTTTGGGACACCTGCT

## Data Availability

Not applicable.
